# Genome sequence of *Clostridium septicum* strain WW106, isolated from influent wastewater at a research center with multiple-species research animal facilities

**DOI:** 10.1128/MRA.00768-23

**Published:** 2023-12-08

**Authors:** Sharon X. Wang, Miseon Park, Christine V. Summage-West, Mariela Reyna, Sung Guk Kim

**Affiliations:** 1 Surveillance/Diagnostic Laboratory, Office of Scientific Coordination, National Center for Toxicological Research, U.S. Food and Drug Administration, Jefferson, Arkansas, USA; 2 Division of Microbiology, National Center for Toxicological Research, U.S. Food and Drug Administration, Jefferson, Arkansas, USA; University of Maryland School of Medicine, Baltimore, Maryland, USA

**Keywords:** *Clostridium septicum*, genome sequence, gangrene, wastewater

## Abstract

*Clostridium septicum* is an anaerobic Gram-positive rod-shaped bacterial pathogen known as a lethal causative agent of progressive gas gangrene in animals and humans. We report the 3.43-Mbp genome sequence of *C. septicum* strain WW106, isolated from influent wastewater at a research center with multiple-species laboratory animal facilities.

## ANNOUNCEMENT

*Clostridium septicum* is an obligatory anaerobic Gram-positive rod-shaped bacterial pathogen that causes a fatal clinical condition associated with malignancies or gas gangrene in animals and humans (1 – 3). Nontraumatic clostridium myonecrosis cases with *C. septicum* shows higher mortality than with *C. perfringens* (4). *C. septicum* produces four toxins (a, b, d, g) and α-toxin, a β-channel forming cytolysin, is known to be responsible for necrotization (1, 2). A cormparative analysis of *C. septicum* genomes revealed that *C. septicum* genomes are variable in prophage composition, CRISPR regions, and restriction-modification systems unlike phylogenetically related *C. chauvoei* genomes that are invariable (1). This complete genome sequence of *C. septicum* expands the current limited genome database and provides better understanding genetics of the fatal human and animal pathogen.

Raw influent wastewater samples (2 × 250 mL) were collected from a research center with multiple-species laboratory animal facilities in Arkansas. The wastewater samples were directly streaked on Remel TSA blood agar and cultured anaerobically (85% N_2_, 10% H_2_, 5% CO_2_) at 37°C for 24 h. A large swarming, mucoid, hemolytic Gram-positive rod-shaped bacterium was identified as *C. septicum* using matrix-assisted laser desorption/ionization-time-of-flight (MALDI-TOF) Biotyper (Bruker, Billerica, MA).

Genomic DNA was isolated from a single colony, cultured anaerobically at 37°C for 24 h, using the Blood & Tissue Kit (Qiagen, Redwood City, CA), and the same DNA was used for Illumina short-read and Nanopore long-read sequencing with no shearing or size-selection. DNA quality and quantity were determined using Nanodrop and Qubit (Fisher Scientific, Waltham, MA). Default parameters were used for all software unless otherwise noted. A short-read genomic library was constructed using the Nextera XT library prep kit and sequenced using the MiSeq v3 reagent kit (2 × 300 bp) on MiSeq (Illumina, San Diego, CA). For long reads, a genomic library was constructed using the ligation sequencing kit SQK-LAK114 and sequenced on Nanopore MinIon FLO-MIN114 R10. The reads were base called using MinKnow 22.12.5 (Guppy6.4.6) on MinION Mk1C (Oxford Nanopore, Oxford, UK). Raw short reads were quality checked and trimmed using Fastq Utilities (CutAdapt v2.2 with Python v3.7.9) in PATRIC (5), and raw long reads were quality checked and trimmed with Porechop v0.2.1 and Galore v0.6.7 in Galaxy (6). Illumina short reads (*n* = 852,034) and Nanopore long reads (*n* = 51,996; *N*_50_ = 3,890 bp) were assembled in two circular contigs with 129 × depth using Unicycler v0.4.8 (default normal bridging mode, rotation) in PATRIC (5). The genome of *C. septicum* WW106 is 3,444,779 bp in length with a GC content of 27.86%, consisting of one circular chromosome of 3,439,472 bp and one circular plasmid of 5,367 bp.The WW106 genome annotated using the NCBI Prokaryotic Genome Annotation Pipeline (PGAP v4.11) has 3,210 coding genes; 1,054 pseudo genes; 87 tRNAs; 19 rRNAs; and 1 CRISPR array (7). The closest type strain genome is *C. septicum* strain DSM 7534 (CP023671) with an Average Nucleotide Identity (ANI) of 99.74% as estimated using MinHash v2.3 (8). The WW102 genome carries a gene predicted to encode α-toxin with >99% identity to *C. septicum* DSM 7534 α-toxin (D17668.1).

## Data Availability

The whole-genome sequence has been deposited at DDBJ/ENA/GenBank under the accession numbers CP131445-CP131446. The raw reads have been deposited in the Sequence Read Archive (SRA) under the accession numbers SRR25462605-SRR25462606.
